# Executive Summary of the Joint Position Paper on Renal Denervation of the Cardiovascular and Interventional Radiological Society of Europe (CIRSE) and the European Society of Hypertension (ESH)

**DOI:** 10.1007/s00270-016-1467-2

**Published:** 2016-09-22

**Authors:** Jonathan G. Moss, Anna-Maria Belli, Antonio Coca, Michael Lee, Giuseppe Mancia, Jan H. Peregrin, Josep Redon, Jim A. Reekers, Costas Tsioufis, Dierk Vorwerk, Roland E. Schmieder

**Affiliations:** 1 Interventional Radiology Unit, North Glasgow University Hospitals, Gartnavel General Hospital, 1053 Great Western Road, Glasgow, G12 0YN UK; 2 Department of Radiology, St George’s Hospital, London, SW17 0QT UK; 3 Hypertension and Vascular Risk Unit, Department of Internal Medicine, Hospital Clínic (IDIBAPS), University of Barcelona, Barcelona, Spain; 4 Department of Radiology, Beaumont Hospital, Dublin 9, Ireland; 5 University of Milano-Bicocca, Milan, Italy; 6 Department of Diagnostic and Interventional Radiology, Institute for Clinical and Experimental Medicine, Prague 4, Czech Republic; 7 Research Institute INCLIVA, University of Valencia and CIBERObn, ISCIII, Madrid, Spain; 8 University of Amsterdam, Department of Radiology, Academic Medical Centre, Amsterdam, The Netherlands; 9 Hippokration Hospital, National and Kapodistrian University of Athens, Athens, Greece; 10 Institute of Radiology, Klinikum Ingolstadt, Ingolstadt, Germany; 11 Nephrology and Hypertension, University Hospital Erlangen, Erlangen, Germany

## Introduction

 This joint position paper, composed by an author group of members of the Cardiovascular and Interventional Radiological Society of Europe (CIRSE) and the European Society of Hypertension (ESH), is being published jointly in the Cardiovascular and Interventional Radiology Journal and the Journal of Hypertension. The paper attempts to review the evidence and provide some guidance and forward direction for this new and potentially still valuable technique. The article presented here is a brief executive summary of the full paper which can be found on the CIRSE and ESH websites.

## Methodology

 CIRSE and the ESH produced this joint position paper using the following process. The formal decision of the two societies to draft a multidisciplinary joint position paper was taken in November 2013, following the discussion on the potential benefits of a joint statement on the occasion of the CIRSE annual meeting 2013 in Barcelona.

Both societies identified and nominated recognised experts as members of the joint writing group for the document. In the case of CIRSE, the renal denervation task force, an already established group of senior interventional radiologists and CIRSE members with significant experience in performing renal artery denervation, represented the society in the working group. In the case of ESH, the eminent members of the joint writing group were selected by the society’s council, based on their expertise.

In early 2014, the first writing workshop was held and a timeline for the drafting process was agreed upon. In the period following the workshop, it was announced that the results of the pivotal HTN-3 regulatory trial would soon be published. Therefore, a new timeline was devised to be able to take into account these important results.

Once HTN-3 had been published, a critical review of the currently available position statements, peer-reviewed articles and regulatory documents in the field of renal denervation was performed with regard to methodology, results and conclusions. Several further drafting workshops and teleconferences were held between the two groups to discuss interpretations and plan the writing phase.

The drafting process of this joint position paper allowed for an extensive exchange among interventional radiologists and hypertension specialists, and achieved a consensus document agreeable to all the contributors. However, the negative results of the first randomised controlled trial with sham control of this therapy that were published during the drafting process had a significant impact on the ongoing assessment. The publication of this position paper intervenes at a point in time where renal artery denervation seems to have lost its momentum in Europe, but the joint writing group deems it thus all the more important to give a comprehensive account of this therapy, including the potential benefits and urgent need for more scientific data of randomised trials.

## Review of Content

Renal denervation (RDN) was reported as an exciting new development for the treatment of resistant hypertension in 2009 [1]. This minimally invasive technique gained rapid acceptance across the globe, although the majority of procedures were carried out in one country (Germany). The Symplicity HTN-2 randomised trial [2] added further supportive evidence (both efficacy and safety) and by late 2013 no fewer than 60 companies were investing in the technology. The global potential is obvious with 5–10 % of hypertensive patients (a third of the world population) falling into the “resistant” category. They are a very high-risk group, and RDN has the potential to result in a marked reduction in cardiovascular morbidity and mortality.

The Symplicity HTN-3 FDA regulatory randomised trial, which would prove pivotal in the assessment of the therapy, included a sham arm and used ABPM in the largest trial to date (*n* = 535). Therefore, it addressed many of the shortcomings of the earlier trials. The results published in NEJM in March 2014 showed a failure to achieve the primary efficacy outcome in reducing office blood pressure at 6 months compared to the sham procedure. The safety endpoint was met with a major adverse event rate of 1.4 % [3].

It is difficult to exaggerate the fallout from this trial and its effect across the globe was almost immediate and perhaps also exaggerated. There has been much criticism and praise of HTN-3 and a wide range of opinions persist. Although RDN appears to be consistently safe across all trials (albeit with limited short-term follow-up), the main controversy is with regard to efficacy and here the trials have produced mixed and conflicting results. As a result of HTN-3 in particular, there has been a dramatic reduction in the use of RDN in all countries in the order of 80 %, and two major companies have withdrawn from the market.

The trial was well designed but has been criticised in several respects. One hundred and eleven different interventionists treated the 364 patients in the active arm (34 % of operators only carried out a single procedure). The majority of patients (*N* = 253) did not have a successful four-quadrant ablation [4]. It must, however, be noted that the guidance technologies applicable to RDN (for example ultrasound) continue to evolve and may offer a more effective denervation in the future.

The interpretation of results also proved challenging. The anatomical studies of human renal nerve anatomy are limited and inconsistent, and further research is needed to guide RDN devices for the future [5, 6]. RDN is currently severely hampered by having no easy method to measure the completeness of denervation, which largely remains a “blind technique”. Variations in the use of aldosterone antagonists drug turbulence in HTN-3 may have introduced confounders.

There are lessons to be learned from previous RDN trial designs and this group remains interested in RDN, although high-quality research is needed before widespread adoption of this expensive technology. This research should include sham arms, use of ABPM to select and monitor patients, objective assessment of drug adherence and long-term assessment of the renal artery and renal function. These trials should be conducted in high-volume specialist centres with the appropriate physician expertise in hypertension management supported by well-trained and audited interventionists. Participation in clinical trials and registries is strongly recommended.
